# Measurement and Decoupling of Hygrothermal-Mechanical Effects with Optical Fibers: Development of a New Fiber Bragg Grating Sensor

**DOI:** 10.3390/s25041037

**Published:** 2025-02-09

**Authors:** Pietro Aceti, Lorenzo Calervo, Paolo Bettini, Giuseppe Sala

**Affiliations:** Department of Aerospace Science and Technology, Politecnico di Milano, Via La Masa n.34, 20156 Milano, Italy

**Keywords:** moisture detection, fiber bragg grating (FBG) sensors, hygrothermal-mechanical effects, bragg equation, structural health monitoring (SHM), health and usage monitoring systems (HUMSs), optical fiber sensing, composite materials in aerospace, temperature and humidity decoupling, predictive maintenance in composite structures

## Abstract

Composite materials are increasingly used in the aviation industry for various aircraft components due to their lightweight and mechanical performances. However, these materials are susceptible to degradation due to environmental factors such as hot–wet environments and freeze–thaw cycles, which can compromise their performance and safety over time. This study develops an innovative Fiber Bragg Grating (FBG) sensor system capable of not only measuring but also decoupling the simultaneous effects of temperature, humidity and strain. Unlike existing FBG systems, our approach integrates a novel theoretical framework and sensor configuration that accurately isolates these parameters in an epoxy resin material. The system incorporates three FBG sensors: one for temperature, one for temperature and humidity and a third one for all three factors. A theoretical framework based on linear strain superposition and constitutive laws was developed to isolate the individual contributions of each factor. Experimental validation in controlled hygrothermal conditions demonstrated the system’s ability to accurately detect and decouple these effects, enabling the monitoring of moisture absorption and composite degradation over time. The proposed system provides a reliable, lightweight and efficient solution for the long-term monitoring of composite structures in extreme conditions. Additionally, it enhances predictive maintenance by improving the accuracy of Health and Usage Monitoring Systems (HUMSs) and provides a method to correct data inconsistencies in already installed sensors, further extending their operational value.

## 1. Introduction

Composite materials have become increasingly prominent across various industrial sectors in recent years, with particular emphasis on their application in the aerospace industry. These materials provide notable advantages, including low weight; high strength; superior stiffness and appreciable resistance to corrosion, impact and fatigue. Such features contribute to reduced aircraft weight and improved overall performances [1,2,3]. Nevertheless, when subjected to adverse environmental conditions, such as significant temperature variations or elevated levels of humidity, composite materials may experience degradation in their mechanical, physical, thermal and electrical properties. Such degradation can critically impair operational performances and affect the service life of aircraft [4,5,6]. Numerous severe aviation incidents [7,8] have revealed that the components most susceptible to moisture ingress are those manufactured using sandwich panels and adhesive-bonded joints, including rudders, ailerons, flaps and sections of the fuselage. These components often lack complete insulation from the external environment due to cracks in the insulating paints caused by minor impacts or paint degradation. Additionally, moisture infiltration frequently occurs at interfaces between different parts [4,9,10]. Both temperature and moisture exert detrimental effects on composites; as a matter of fact, the mechanical properties of the matrix are degraded by high temperatures, while fiber-dominated properties are primarily affected by low temperatures. Moisture affects both the matrix and the fibers, owing to their hygroscopic nature. In Glass-Fiber-Reinforced Polymer (GFRP) and Carbon-Fiber-Reinforced Polymer (CFRP) composites, where epoxy resins are often used, the primary effects are observed in the matrix, as moisture acts as a plasticizer, disrupts Van der Waals bonds, breaks cross-links within the polymeric chains and induces leaching issues [4]. In the case of natural fibers, such as flax fibers [11,12], the reinforcement itself loses its mechanical properties, thereby compromising the overall material properties. High temperatures further exacerbate moisture absorption, weakening the matrix’s mechanical properties, a hot–wet condition being the most critical one [13,14,15]. Consequently, it is essential to continuously monitor the hygrothermal effects in composite structures to mitigate severe damage and optimize periodic maintenance schedules.

Optical-fiber-based HUMS/SHM systems are widely employed in the aerospace sector due to their low cost, high accuracy, high sensitivity, immunity to electromagnetic jamming and their capabilities for multiplexing and remote sensing. Additionally, their small size allows these sensors to be easily embedded within composite panels, enabling real-time monitoring of internal structural deterioration and the development of new maintenance techniques [16,17]. In [18], it is shown that optical fibers coated with hydrophilic materials, such as polyimide, are sensitive to relative humidity. This sensitivity introduces an additional variable that must be considered, as humidity can compromise the accuracy of temperature and strain measurements. Therefore, without the precise monitoring of humidity, the sensor’s measurements risk becoming unreliable.

Various techniques have been developed over time to measure humidity in composite structures: these methods include electrical, mechanical and optical sensors [19,20,21,22]. However, in aerospace applications, weight is a critical parameter, and adding extra sensors and interrogation units can increase system complexity and overall weight. To address this challenge, the present study focuses on the use of Fiber Bragg Grating (FBG) sensors, which can utilize the hygroscopicity of the polyimide coating to measure humidity. This approach allows the integration of humidity sensing into the existing optical fiber system without introducing additional systems.

### FBG Sensors and the Bragg Law

Sensors inscribed within uncoated optical fibers can measure temperature and strain. A change in temperature or in a state of stress causes a change in the reflected peak due to a variation of the period of the grating and to a variation of the effective refractive index of the glass [23]. Due to their intrinsic fragility, optical fibers are often coated with polymer layers to protect them during installation, or when embedded in composite materials, during the manufacturing phase. If the optical fiber is coated with a hygrophilic material or embedded in a hygroscopic resin system, the sensor will also be sensitive to relative humidity due to the swelling of the coating [18,24,25] and of the resin system. In order to obtain accurate measurements, it is necessary to measure and decouple the wavelength shift contributions due to mechanical and hygrothermal loads.

The relationship between the Bragg wavelength, the effective refractive index and the grating period is given by Equation (1):

(1) $$\lambda_{B}=2n_{eff}\Lambda$$

where $\lambda_{B}$ is the Bragg wavelength, $n_{eff}$ is the effective refractive index and Λ is the period of the grating. To exploit FBG sensors for temperature, relative humidity and deformation sensing, a relationship between the Bragg wavelength and these quantities must be established. As far as naked optical fibers are concerned, they are sensitive only to temperature and strain. The relationship can be derived from Equation (1), leading to the following linear form [18,26]:

(2) $$\frac{\Delta \lambda_{B}}{\lambda_{B}}=\left(1-p_{e}\right)\varepsilon_{m}+\left(\alpha_{g}+\xi \right)\Delta T$$

where $p_{e}$ is the photo-elastic coefficient, $\alpha_{g}$ is the coefficient of thermal expansion of the glass and ξ is the thermo-optic coefficient. As a consequence, $K_{m}=\left(1-p_{e}\right)$ and $K_{T}=(\alpha_{g}+\xi$) represent the sensitivities to strain and temperature.

To account for the moisture effect, the fiber must be coated with a hydrophilic material, such as polyimide, which is one of the most used coating in the aeronautical field. Consequently, a new linear term must be introduced in Equation (2), leading to:

(3) $$\begin{array}{c} \begin{array}{cc} \frac{\Delta \lambda_{B}}{\lambda_{B}} & =K_{m}\varepsilon_{m}+K_{T}\Delta T+K_{RH}\Delta RH \end{array} \end{array}$$

where $K_{RH}$ is the sensitivity to relative humidity. This quantity will be analyzed in Section 2. The aim of this study is to provide a modified version of Bragg’s law capable of identifying the mechanical and hygrothermal effects on a system composed of fiber, coating and bulk resin. A new sensor will be introduced, designed to measure and decouple temperature (*T*), mechanical strain (ε) and relative humidity (RH).

## 2. Theoretical Formulation

A typical coated optical fiber consists of three main components: a glass core, a cladding made of another type of glass with a slightly lower refractive index than the core and an external coating. The coatings most used in the aeronautical field are Polyimide and Ormocer ^®^ (i.e., ORganic MOdified CERamic). They are characterized by excellent adhesion between the fiber and the coating; in fact, the latter must be able to transfer the load to the fiber core where the sensor is inscribed. Moreover, optical fibers can be embedded into a host material for several reasons, e.g., to measure strain at specific internal points of interest and to better protect the fiber from impact or harsh environmental conditions.

**Figure 1:**
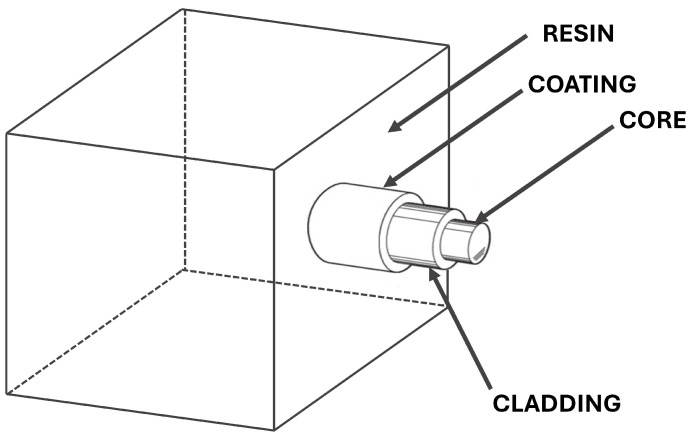
A typical optical fiber structure.

A typical Representative Volume Element (RVE) is presented in Figure 1, where the optical fiber is embedded into a cube of bulk resin. When an optical fiber embedded in bulk resin is simultaneously subjected to temperature, mechanical strain/stress and humid environment, both the fiber, the coating and the bulk resin contribute to the overall normalized wavelength shift. As demonstrated in previous studies [18], the contribution to the normalized Bragg wavelength shift is as follows:

(4) $$\frac{\Delta \lambda }{\lambda_{00}}=\varepsilon_{tot}+\xi \Delta T-p_{e}\left(\varepsilon_{res}+\varepsilon_{m}\right)$$

where:

- $\frac{\Delta \lambda }{\lambda_{00}}$ is the normalized Bragg wavelength shift. $\lambda_{00}$ is the sensor response at 0 °C and 0% of relative humidity. The value of $\lambda_{00}$ is arbitrary and this specific choice was made for convenience;
- $\varepsilon_{tot}$ is the actual strain affecting the glass core, which takes into account, according to the law of mixtures, the different contributions due to glass, coating and bulk resin. It takes into account the total strain given by thermal, hygroscopic and mechanical contributions;
- ξΔT is the contribution to the normalized wavelength’s shift given by the change of $n_{eff}$ due to temperature variation;
$-p_{e}\left(\varepsilon_{res}+\varepsilon_{m}\right)$ is the contribution to the normalized wavelength’s shift due to the change of $n_{res}$, which comes from a strain field generated by external or residual stress;
- $\varepsilon_{res}$ is the strain related to the residual stress generated according to the mixture laws. In fact, due to the compatibility equations, in a system composed of different materials, they must all experience the same strain; this will generate a different stress field in each material and—consequently—some residual stresses. This is always verified when the two-phase material is modeled by two springs in parallel and when perfect adhesion between the phases is assumed;
- $\varepsilon_{m}$ is the strain generated by an external force.

The contributions just presented will be quantified for a coated optical fiber embedded in bulk resin using a micro-mechanical approach in the following section, Section 2.1. The final form is presented in Section 2.2.

### 2.1. Micro-Mechanics Approach

Let us consider a scenario where a coated optical fiber is embedded into a bulk resin system, simultaneously subjected to both mechanical and hygrothermal loads. The following quantities are defined, noting that the subscript “*g*” refers to glass (i.e., core and cladding), “*c*” refers to the coating and “*r*” refers to the resin:

- $E_{g},A_{g},V_{g}=\left(\frac{A_{g}}{A_{tot}}\right)$ are, respectively, the Young modulus, the section area and the volumetric fraction of the glass;
- $E_{c},A_{c},V_{c}=\left(\frac{A_{c}}{A_{tot}}\right)$ are, respectively, the Young modulus, the section area and the volumetric fraction of the coating;
- $E_{r},A_{r},V_{r}=\left(\frac{A_{r}}{A_{tot}}\right)$ are, respectively, the Young modulus, the section area and the volumetric fraction of the resin;
- $\alpha_{g}$, $\alpha_{c}$ and $\alpha_{r}$ are, respectively, the CTE of glass, coating and resin.
- $\beta_{c}$ and $\beta_{r}$ are, respectively, the swelling coefficient of the coating, such as $\varepsilon_{RH-c}=\beta_{c}\Delta RH$, and the swelling coefficient of the resin, such as $\varepsilon_{RH-r}=\beta_{r}\Delta RH$;
- $A_{tot}=A_{g}+A_{c}+A_{r}$, $E_{tot}$ are, respectively, the total area and the equivalent Young modulus of the system composed by the optical fiber and coating.

The total force experienced by the system is the sum of hygrothermal and mechanical forces:

(5) $$\begin{array}{cc} F_{tot} & =\left(A_{g}E_{g}\alpha_{g}+A_{c}E_{c}\alpha_{c}+A_{r}E_{r}\alpha_{r}\right)\Delta T+\left(A_{c}E_{c}\beta_{c}+A_{r}E_{r}\beta_{r}\right)\Delta RH+F_{ext}=\\ & =F_{g}^{T}+F_{c}^{T}+F_{r}^{T}+F_{c}^{RH}+F_{r}^{RH}+F_{ext} \end{array}$$

where $F_{ext}$ is an external axial force along the fiber axis. According to the mixture law and compatibility equations, it results in:

(6) $$\left\{\begin{array}{c} \varepsilon_{tot}=\varepsilon_{c}=\varepsilon_{g}=\varepsilon_{r}\\ F_{tot}=F_{g}+F_{c}+F_{r} \end{array}\right.$$

where $F_{c}$, $F_{g}$ and $F_{r}$ are, respectively, the portion of load borne by the coating, the glass (core and cladding) and the resin. Manipulating the above Equation (6), we get:

(7) $$\left\{\begin{array}{c} \varepsilon_{tot}=\varepsilon_{c}=\varepsilon_{g}=\varepsilon_{r}\\ \frac{F_{tot}}{A_{tot}}=\frac{F_{g}}{A_{tot}}\frac{A_{g}}{A_{g}}+\frac{F_{c}}{A_{tot}}\frac{A_{c}}{A_{c}}++\frac{F_{r}}{A_{tot}}\frac{A_{r}}{A_{r}}\to \sigma_{tot}=V_{c}\sigma_{c}+V_{g}\sigma_{g}\;+V_{r}\sigma_{r} \end{array}\right.$$

By solving the system in Equation (7), it is possible to determine the equivalent Young modulus of the system:

(8) $$E_{tot}\varepsilon_{tot}=V_{c}E_{c}\varepsilon_{c}+V_{g}E_{g}\varepsilon_{g}+V_{r}E_{r}\varepsilon_{r}\to E_{tot}=V_{c}E_{c}+V_{g}E_{g}+V_{r}E_{r}$$

This represents the Young modulus of the whole system, composed of glass optical fiber, coating and bulk resin. Assuming a linear elastic constitutive law, actual strain assumed by the system can be computed as:

(9) $$\varepsilon_{tot}=\frac{F_{tot}}{A_{tot}E_{tot}}\to \varepsilon_{tot}=\frac{F_{tot}}{A_{c}E_{c}+A_{g}E_{g}+A_{r}E_{r}}$$

Therefore, the strain becomes:

(10) $$\begin{array}{cc} \varepsilon_{tot}= & \frac{F_{tot}}{A_{tot}E_{tot}}=\\ = & \frac{A_{g}E_{g}\alpha_{g}\Delta T+A_{c}E_{c}\alpha_{c}\Delta T+A_{r}E_{r}\alpha_{r}\Delta T}{A_{c}E_{c}+A_{g}E_{g}+A_{r}E_{r}}+\\ & +\frac{A_{c}E_{c}\beta_{c}\Delta RH+A_{r}E_{r}\beta_{r}\Delta RH+F_{ext}}{A_{c}E_{c}+A_{g}E_{g}+A_{r}E_{r}}=\\ = & \frac{A_{g}E_{g}\alpha_{g}+A_{c}E_{c}\alpha_{c}+A_{r}E_{r}\alpha_{r}}{A_{g}E_{g}+A_{c}E_{c}+A_{r}E_{r}}\Delta T+\\ & +\frac{A_{c}E_{c}\beta_{c}+A_{r}E_{r}\beta_{r}}{A_{g}E_{g}+A_{c}E_{c}+A_{r}E_{r}}\Delta RH+\\ & +\frac{F_{ext}}{A_{tot}E_{tot}} \end{array}$$

Let us now quantify $\varepsilon_{res}$, which is the virtual strain applied to the glass fiber core due to the residual stress. It can be interpreted as the strain that the core would assume after reducing the strain that the core actually assumes. It results:

(11) $$\varepsilon_{res}=\alpha_{g}\Delta T-\left(\frac{A_{g}E_{g}\alpha_{g}+A_{c}E_{c}\alpha_{c}+A_{r}E_{r}\alpha_{r}}{A_{g}E_{g}+A_{c}E_{c}+A_{r}E_{r}}\Delta T+\frac{A_{c}E_{c}\beta_{c}+A_{r}E_{r}\beta_{r}}{A_{g}E_{g}+A_{c}E_{c}+A_{r}E_{r}}\Delta RH\right)$$

Equation (11) shows that, whenever the system composed of glass fibers, coating and resin is subjected to a thermal load, a residual stress is generated. This residual stress, $\sigma_{res}=E_{g}\varepsilon_{res}$, is calculated as the product of the elastic modulus of the glass ($E_{g}$) and the strain ($\varepsilon_{res}$). Such stress will induce a change in the refractive index, quantified through the photoelastic coefficient ($p_{e}$).

### 2.2. Modified Bragg Equation for a Coated Optical Fiber Embedded in Bulk Resin

Now the modified Bragg equation for a coated optical fiber subjected to mechanical and hygrothermal loads can be derived, substituting Equations (10) and (11) into Equation (4):

(12) $$\begin{array}{cc} \frac{\Delta \lambda }{\lambda_{00}}= & \frac{A_{g}E_{g}\alpha_{g}\Delta T+A_{c}E_{c}\alpha_{c}\Delta T+A_{r}E_{r}\alpha_{r}\Delta T}{A_{c}E_{c}+A_{g}E_{g}+A_{r}E_{r}}+\\ & +\frac{A_{c}E_{c}\beta_{c}\Delta RH+A_{r}E_{r}\beta_{r}\Delta RH+F_{ext}}{A_{c}E_{c}+A_{g}E_{g}+A_{r}E_{r}}+\xi \Delta T+\\ & -p_{e}\left[\alpha_{g}\Delta T-\left(\frac{A_{g}E_{g}\alpha_{g}+A_{c}E_{c}\alpha_{c}+A_{r}E_{r}\alpha_{r}}{A_{g}E_{g}+A_{c}E_{c}+A_{r}E_{r}}\Delta T+\right.\right.\\ & \left.\left.+\frac{A_{c}E_{c}\beta_{c}+A_{r}E_{r}\beta_{r}}{A_{g}E_{g}+A_{c}E_{c}+A_{r}E_{r}}\Delta RH\right)+\varepsilon_{m}\right] \end{array}$$

It is worth noting that, thanks to the compatibility equation, $\varepsilon_{m}^{c}=\varepsilon_{m}^{g}=\varepsilon_{m}^{r}=\varepsilon_{m}=\frac{F_{tot}}{A_{tot}E_{tot}}$. By manipulating Equation (12) in order to explicit the external mechanical and hygrothermal loads, the following result is obtained:

(13) $$\begin{array}{cc} \frac{\Delta \lambda }{\lambda_{00}}= & \left[\left(1+p_{e}\right)\;\frac{A_{g}E_{g}\alpha_{g}+A_{c}E_{c}\alpha_{c}+A_{r}E_{r}\alpha_{r}}{A_{g}E_{g}+A_{c}E_{c}+A_{r}E_{r}}-p_{e}\alpha_{g}+\xi \right]\Delta T+\\ & +\left[\left(1+p_{e}\right)\;\frac{A_{c}E_{c}\beta_{c}+A_{r}E_{r}\beta_{r}}{A_{g}E_{g}+A_{c}E_{c}+A_{r}E_{r}}\right]\Delta RH+\\ & +\left(1-p_{e}\right)\;\varepsilon_{m} \end{array}$$

This new formulation allows for the derivation of linear coefficients that link hygrothermal and mechanical loads to the normalized Bragg wavelength shift, given the properties of the constituent materials. For the sake of brevity, Equation (13) can be rewritten as follows:

(14) $$\frac{\Delta \lambda }{\lambda_{00}}=K_{T}\Delta T+K_{RH}\Delta RH+K_{m}\varepsilon_{m}$$

where $K_{T},K_{RH}$ and $K_{m}$ represent, respectively, the sensitivities to temperature, relative humidity and mechanical strain of a coated embedded optical fiber:

(15) $$\begin{array}{cc} & K_{m}=\left(1-p_{e}\right)\\ & K_{T}=\left(1+p_{e}\right)\frac{A_{g}E_{g}\alpha_{g}+A_{c}E_{c}\alpha_{c}+A_{r}E_{r}\alpha_{r}}{A_{g}E_{g}+A_{c}E_{c}+A_{r}E_{r}}+\xi -p_{e}\alpha_{g}\\ & K_{RH}=\left(1+p_{e}\right)\frac{A_{c}E_{c}\beta_{c}+A_{r}E_{r}\beta_{r}}{A_{g}E_{g}+A_{c}E_{c}+A_{r}E_{r}} \end{array}$$

## 3. Manufacturing Process of the Sensor

**Figure 2:**
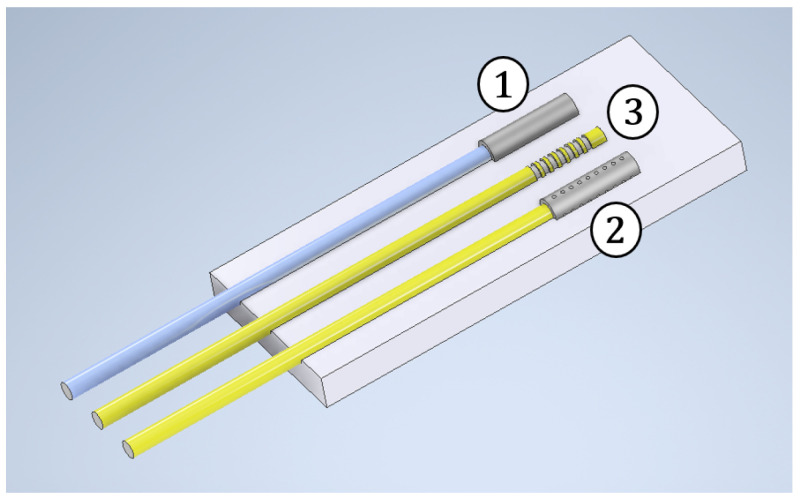
Arrangement of three FBGs in three different fibers to create the sensor.

This paper aims to provide a sensor capable of measuring and decoupling mechanical strain, thermal expansion and hygroscopic swelling through an optical fiber system. In Equation (13), it was shown that temperature, mechanical strain and relative humidity contribute to shifting the normalized Bragg wavelength. To decouple the measurements and obtain the three quantities, a set of three sensors has to be inscribed on the same fiber or on separate fibers, as shown in Figure 2. The use of appropriate capillary tubes and the analysis of the behavior of fibers with/without coating allow us to obtain the three linearly independent measures shown in Equation (16).

The measuring device shown in Figure 2 is made of three elements, each consisting of an FBG sensor inscribed on an optical fiber, in turn embedded in resin as the host material:

- Element #1 consists of an FBG inscribed on an uncoated optical fiber, meaning it is unable to measure the variation of relative humidity. Moreover, it is confined inside a metallic capillary tube, which does not allow any resin-to-sensor load transfer, so as to make it insensitive to mechanical loads. For these reasons, this sensor is able to measure only temperature;
- Element #2 consists of an FBG sensor inscribed on a coated optical fiber and is able to detect both temperature and relative humidity. Moreover, it is confined inside a metallic capillary tube provided with micrometric holes, allowing moisture to diffuse into the cavity to induce coating swelling. At the same time, the stiffness of the metallic capillary tube does not allow external mechanical loads to excite the sensor. For these reasons, this sensor is able to measure relative humidity and temperature but no mechanical strain;
- Element #3 represents the so called “free sensor” and consists of an FBG sensor inscribed on a coated optical fiber embedded in bulk resin. This sensor is able to measure temperature, relative humidity and mechanical strain.

The three equations describing FBG sensors are listed hereafter, where subscripts “1”, “2” and “3” stand for, respectively, capillary sensor, micro-perforated capillary sensor and free sensor:

(16a)Δλ1λ00−1=KT1ΔT(16b)Δλ2λ00−2=KT2ΔT+KRH2ΔRH(16c)Δλ3λ00−3=KT3ΔT+KRH3ΔRH+Kε3εm

where $\lambda_{i}$ represents the Bragg wavelength reflected by the sensor, $K_{x_{i}}$ are the sensitivities (from FBG sensors characterization tests) and $\lambda_{00-i}$ is the value of the sensor’s response at 0 °C and 0% relative humidity. It cannot be a measured value since it is impossible to reach 0 °C and 0% relative humidity in a climatic chamber due to Mollier’s diagram. As a matter of fact, this value was derived by computing the y-intercept of the calibration curves of the each sensor. The only remaining unknowns are ΔT, ΔRH and Δε, the three quantities to be provided by a device consisting of a system of three independent sensors. The realization of the three sensors is presented hereafter.

### 3.1. Thermal Measurements

**Figure 3:**
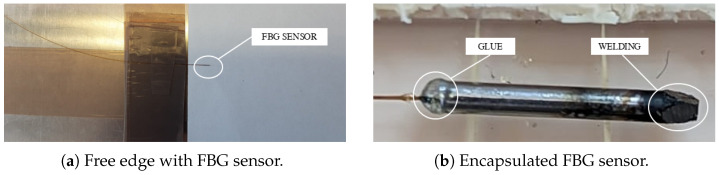
Manufacturing of the thermal sensor.

The first sensor must be sensitive to temperature only, to directly provide its value. In order to do so, a free sensor, with a gauge length of 3 mm, is encapsulated inside a metallic capillary tube $d_{tube}=1.7$ mm in diameter and 16 mm in length, as seen in Figure 3 [27,28]. The tube is welded at one edge and bonded at the other. Strains cannot affect the FBG because the sensor is isolated inside a stiff component and the fiber is insensitive to relative humidity since the coating has been removed from the sensing region.

### 3.2. Hygrothermal Measurements

In order to allow humidity sensing in addition to temperature sensing, a coated fiber must be used. However, the sensor must still be isolated from mechanical effects. To this end, the FBG sensor, with a gauge length of 3 mm, is encapsulating inside a metallic capillary tube, as in the previous case, but equipped with several holes. Thus, the temperature can be measured as well and the moisture can diffuse inside the coating through the holes. The capillary tube is $d_{tube}=1.7$ mm in diameter and 21 mm in length, and the holes are $d_{holes}=0.3$ mm in diameter. This latter value was designed so that the resin fills the holes in the tube’s external wall but does not flow inside the tube’s internal volume, thanks to surface tension. In fact, if the resin were to enter, it would mechanically connect the capillary to the sensor.

**Figure 4:**
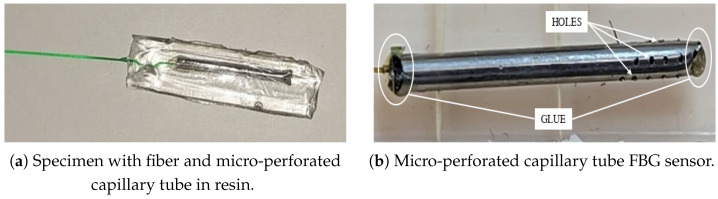
Realization of the micro-perforated capillary tube FBG sensor.

A test was performed to investigate the behavior of the resin–holes interaction. A fiber was inserted into a micro-perforated capillary tube and then drowned in epoxy resin (Figure 4a).

**Figure 5:**
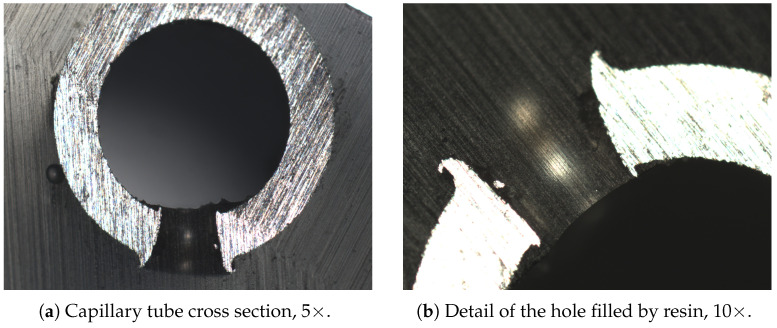
Optical microscope analysis.

Once the resin had solidified, the specimen cross-section was analyzed through optical microscopy (Figure 5). Figure 5b shows that the resin completely filled the hole, but not the internal volume of the capillary tube. Therefore, the coated fiber is exposed to relative humidity (diffused by the epoxy resin through the holes and by the air inside the tube) without being in direct contact with the resin. As a matter of fact, any bonding between the fiber and the resin would compromise the correct swelling of the coating, thus inducing undesired residual stresses.

To guarantee an homogeneous moisture absorption, a total of 20 holes were made in the outer wall of the capillary tube (Figure 4b). As before, the fiber was inserted into the micro-perforated metallic tube and was bonded at one extremity to seal off the internal region.

### 3.3. Hygrothermal-Mechanical Measurements

A completely free FBG sensor inscribed inside a coated fiber was used. No metallic capillary tubes were adopted: the sensor was ready to be assembled.

### 3.4. Specimen Manufacturing

**Figure 6:**
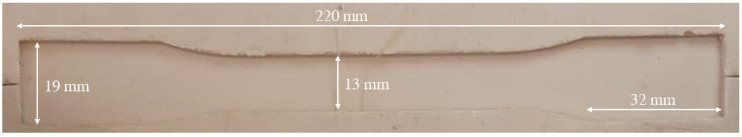
Silicone mold.

To test the capability of the device, made of three FBG sensors, to decouple temperature, relative humidity and mechanical strain, a bulk resin specimen was produced. The silicon mold (Figure 6) was 220 mm long, 19 mm (clamping region) and 13 mm (measuring portion) wide and 3 mm thick.

A bi-component PROCHIMA epoxy resin was used, having a $\rho_{resin}=1120\;\mathrm{kg}/m^{3}$ density and a 1600–1800 mPas range viscosity.

**Figure 7:**
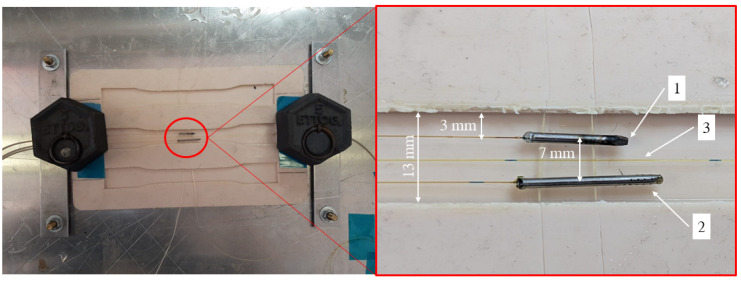
Detail of the sensor arrangement and assembly process.

Figure 7 shows the final result. The three FBG sensors were positioned in the measuring region, side by side, at mid-plane. Figure 7 shows the upper sensor (sensitive to temperature (capillary), the central sensor (completely free) and the lower sensor (affected by relative humidity and temperature (micro-perforated capillary). Once in position, the optical fibers were inserted into thicker sheaths to protect them from breakage during the production process. Finally, the epoxy resin was cast into the mold until completely filled. Once the resin was fully cured, the sensor could be removed from the silicone mold.

## 4. Results and Discussion

To assess the sensor correct functioning and validate the modified Bragg law obtained in Equation (13), a conditioning test was carried out in a climate chamber according to ASTM standard D5529/D5529M [29]. The climate chamber was an ACS DY110C, while the optical interrogator was a Micron Optics sm130. The sensorized specimen, immersed in a water vessel, was closed inside the climate chamber, whose temperature was set to 75 °C to enhance specimen moisture uptake. The humidity controller of the climate chamber was disabled since the specimen was immersed in water. No mechanical load was applied. The sensors embedded into the specimen were connected to an interrogator collecting data. The test lasted 20 days and was considered concluded once the signals from the three sensors reached an asymptotic value. Together with the sensorized specimen, another five specimens, made with the same resin, were immersed in the water vessel, and their weight was monitored time-by-time. The latter measurements were used to quantify mass gain due to resin moisture uptake. Such data will be used to assess the resin swelling coefficient (Section 4).

**Figure 8:**
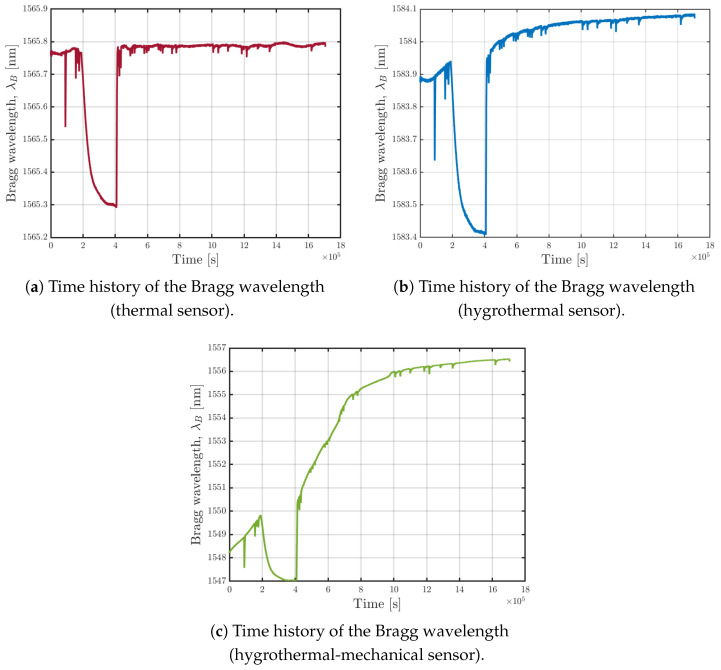
Time history of the Bragg wavelength for the three sensors.

Figure 8 presents the time evolution of Bragg wavelength (three sensors) and shows an abrupt drop occurring mid-experiment due to a temporary stop of the climate chamber’s operation, which took place between $2\times 10^{5}$ s and $4\times 10^{5}$ s. Such an interruption highlights the sensors capability to detect temperature variations and further demonstrates that absorption rate is dependent on temperature.

Figure 8a shows the trend measured by the sensor isolated inside the capillary tube. As expected, the trend remains constant as it is sensitive only to temperature, which was maintained constant inside the climate chamber. The spikes observed are caused by temperature drops due to the climate chamber being opened to weigh the non-sensorized specimens.

Figure 8b reports the trend measured by the sensor encapsulated in the micro-perforated capillary tube. It shows a slight increase due to moisture uptake within the resin specimen, which is a diffusion phenomenon, thus occurring very slowly. This sensor is also sensitive to temperature; therefore, the drop due to the suspension of the climate chamber’s operation is noticeable, as are the temperature drops due to the opening of the climate chamber door.

A similar trend is shown in Figure 8c, where the free sensor is sensitive to all the quantities. As a matter of fact, lacking external loads, the behavior of this sensor is comparable to that of the sensor specifically designed to measure only temperature and relative humidity.

Now, temperature, relative humidity and deformation can be determined by analyzing the respective Bragg wavelength variations according to Equation (12) and by computing the normalized Bragg wavelength shift according to:

(17) $$\frac{\Delta \lambda }{\lambda_{00}}=\frac{\lambda -\lambda_{00}}{\lambda_{00}}$$

where $\lambda_{00}$ is the value of the sensor’s response at 0 °C and 0% relative humidity. Other numerical values used in the equation are listed in Table 1. These values are derived from the datasheet of the optical fiber and the resin used in the system, from standard experiments, literature sources [24,26,30,31,32,33] and from a calibration study of the same optical fiber reported in [18].

**Figure 9:**
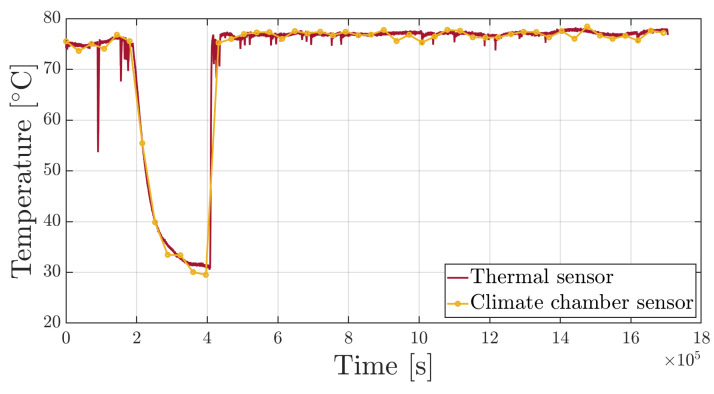
Time history comparison between the temperature measured by the optical thermal sensor and the sensor of the climate chamber controller.

For this purpose, the specimen’s dimensions and the resin parameters need to be accurately measured. The dimension of specimens comply to ASTM D638-14 [34] standard requirements for type I specimens. The resin cross-section ($A_{r}$) was computed by subtracting the areas of the two capillary tubes from the total cross-section area of the specimen. The resin’s Young’s modulus was measured through tensile tests carried out on identical specimens according to ASTM D638-14 [34]. Figure 9 shows the temperature trend obtained by solving the first equation of System (16) compered to the temperature measured by the climate chamber. The fiber designated to measure temperature is not loaded due to the presence of the capillary and cannot absorb humidity since the coating was removed. The governing equation of the sensor response is:

(18) $$\frac{\lambda_{B}-\lambda_{00-1}}{\lambda_{00-1}}=\left(\xi +\alpha_{g}\right)\Delta T\Rightarrow \Delta T=\frac{\lambda_{B}-\lambda_{00}}{\lambda_{00}\left(\xi +\alpha_{g}\right)}$$

where $\lambda_{00-1}=1564.964315$ nm, while ξ and $\alpha_{g}$ are listed in Table 1.

The temperature values measured by the thermal sensor strongly correlate to the value (75 °C) set by the climate chamber controller, which is also measured by internal thermal sensors that acquire data at hourly intervals.

**Figure 10:**
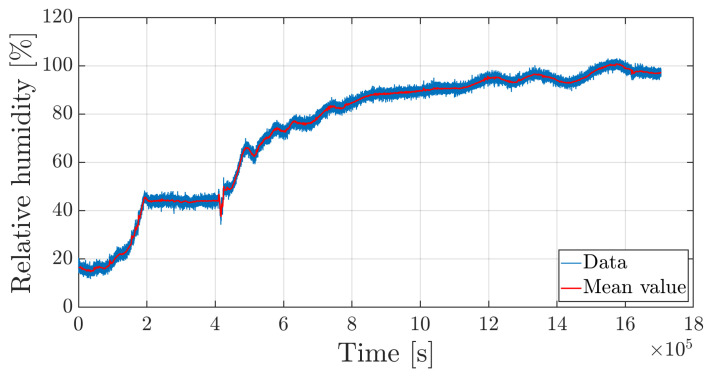
Time history of the relative humidity (hygrothermal sensor).

Once the thermal contribution (ΔT) are known, relative humidity can be derived by removing the thermal contribution from the hygrothermal sensor’s wavelength trend. As a matter of fact, the fiber designated to measure relative humidity is not loaded thanks to the presence of the capillary tube and can measure only temperature and relative humidity. Computing the normalized Bragg wavelength shift, as shown in Equation (17), the following equation can be solved, thus computing the relative humidity variation [18]:

(19) $$\Delta RH=\left\{\frac{\lambda_{B}-\lambda_{00-2}}{\lambda_{00-2}}-\left[\left(1+p_{e}\right)\frac{A_{c}E_{c}\alpha_{c}+A_{g}E_{g}\alpha_{g}}{A_{c}E_{c}+A_{g}E_{g}}-p_{e}\alpha_{g}+\xi \right]\Delta T\right\}\left[\frac{A_{c}E_{c}+A_{g}E_{g}}{\left(1+p_{e}\right)A_{c}E_{c}\beta_{c}}\right]$$

where $\lambda_{00-2}=1582.95895$ nm and all other values are listed in Table 1. Figure 10 shows that relative humidity gradually increases up to an asymptotic value. The curve also shows the average value of the relative humidity trend obtained by filtering out noise. In fact, the sensor is bonded at one edge and cantilevered inside the micro-perforated capillary tube, and so the vibrations induced by the climate chamber compressor and fan alter the signal with noise.

**Figure 11:**
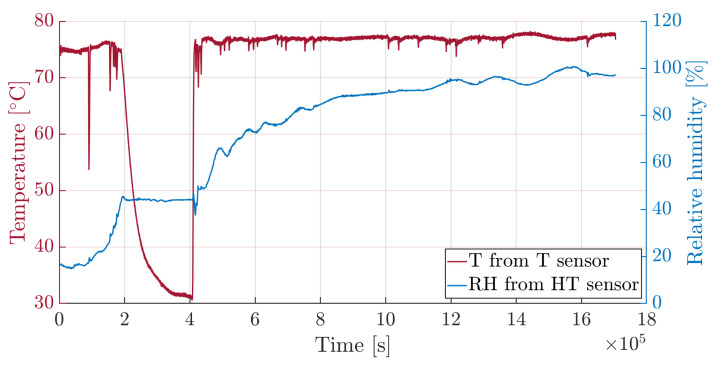
Time histories of temperature and relative humidity.

The superposition of temperature and relative humidity time histories, reported in Figure 11, show that, during the temporary stop of the climate chamber functioning, temperature decreases and the relative humidity remains constant. This phenomenon is explained by the Fick law, where the diffusion coefficient depends on temperature. In fact, while the system is maintained at 75 °C temperature, the absorption process is accelerated and gives rise to a rapid moisture uptake. When the temperature drops, the diffusion coefficient decreases to such an extent that mass gain becomes negligible.

**Figure 12:**
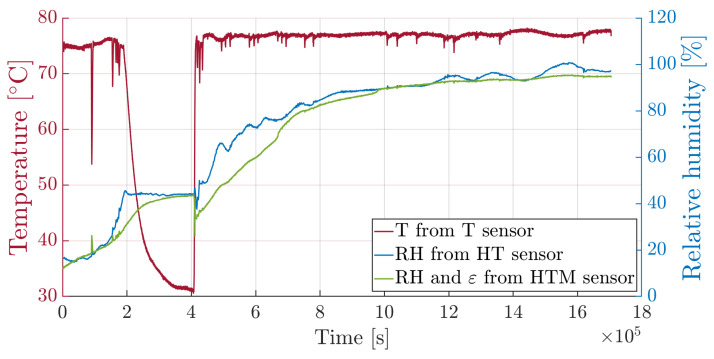
Relative humidity comparison: superposition of the RH time-histories measured by hygrothermal and hygrothermal-mechanical sensors.

Let us now consider the third sensor without a capillary tube. Temperature offset must be removed to obtain the hygro-mechanical contribution, as shown in Equation (19). Since no external loads are applied (i.e., assuming null mechanical strain), the free sensor should be equivalent to the one inside the micro-perforated capillary. Figure 12 reports the superposition of the two relative humidity time-histories, respectively provided by the sensor without capillary and the sensor encapsulated into the micro-perforated capillary, exhibiting substantially similar responses. This leads to a $\beta_{r}=1.636\times 10^{-4}\;\left[1/\mathrm{RH}\right]$ resin swelling coefficient, to be later validated by comparing the swelling measured by the sensor to the swelling generated by the amount of water measured by the conditioned specimen with no sensors embedded.

Until now, mechanical strain is set to null since no external load is applied to the specimen. Considering the third sensor, by solving Equation (13), the hygrothermal contribution to normalized Bragg wavelength shift can be subtracted in order to evaluate the mechanical strain applied to the specimen such as:

(20) $$\begin{array}{cc} \varepsilon_{m}= & \frac{1}{1-p_{e}}\left\{\frac{\Delta \lambda }{\lambda_{00-3}}\right.\\ & -\left[\left(1+p_{e}\right)\frac{A_{g}E_{g}\alpha_{g}+A_{c}E_{c}\alpha_{c}+A_{r}E_{r}\alpha_{r}}{A_{g}E_{g}+A_{c}E_{c}+A_{r}E_{r}}-p_{e}\alpha_{g}+\xi \right]\Delta T\\ & -\left.\left[\left(1+p_{e}\right)\frac{A_{c}E_{c}\beta_{c}+A_{r}E_{r}\beta_{r}}{A_{g}E_{g}+A_{c}E_{c}+A_{r}E_{r}}\right]\Delta RH\right\} \end{array}$$

**Figure 13:**
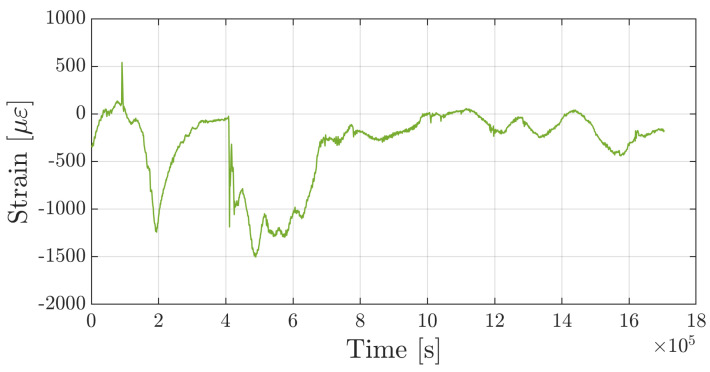
Strain time-history measured by the hygrothermal-mechanical sensor.

Figure 13 shows strain time history measured by the third sensor once both thermal and hygroscopic contributions are excluded. Mechanical deformation cannot be maintained as null during the test because, during conditioning, moisture uptake and high temperature notably degraded the mechanical properties of specimens and favored their bending deformation. At $t=8\times 10^{5}$ s, the specimen was returned to its original configuration and a metallic net was placed at a height slightly above the thickness of the test specimen. So doing, the specimen was no longer able to bend, as shown by the terminal part of the curve (Figure 13), where the strain remained close to zero.

**Figure 14:**
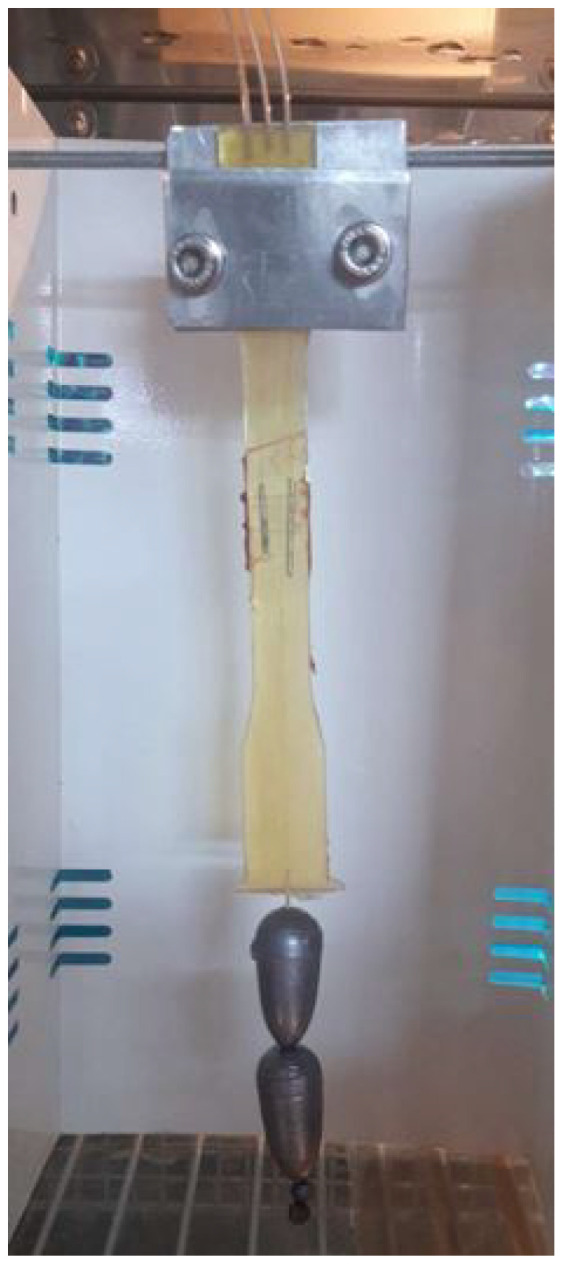
Saturated sensorized specimen at 50 °C temperature loaded by 110 g weights.

An additional test was conducted to further investigate and validate the free sensor response. Once saturation was achieved saturation, the climate chamber was set to maintain 50 °C and the sensorized specimen was constrained vertically, loaded by two 110 g lead weights at the bottom edge, as shown in Figure 14. Considering the specimen’s own weight of 7.52 g, the total system weight was 117.52 g. The signals measured by the three sensors, decoupled as previously described, gave a temperature of 51.7 °C, a relative humidity of 96 % and a total mass of 115.3 g, in close agreement with the real applied mass and assessing sensor effectiveness in detecting mechanical deformations. Therefore, the sensor effectively measures and decouples temperature, relative humidity and strain.

**Figure 15:**
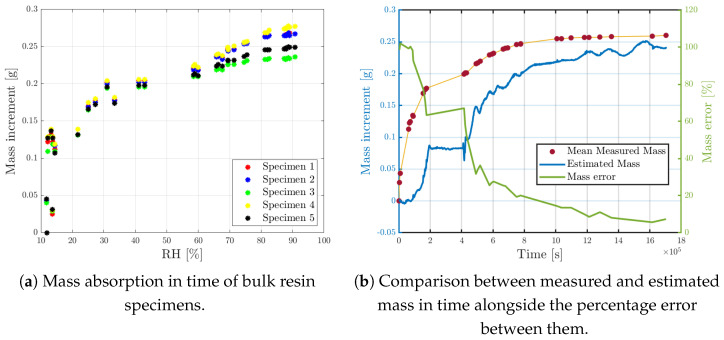
Measured mass from bulk specimens and comparison with estimated mass from the hygrothermal sensor.

Finally, an experimental validation of the resin swelling coefficient ($\beta_{r}$) was carried out. Five specimens were produced using the same resin and the same molds. They were conditioned in the climate chamber together with the sensorized one. They were initially weighed in dry conditions and then time-by-time at regular intervals using a precision scale, to keep track of mass gain due to moisture uptake, as shown in Figure 15a.

Under the hypothesis of isotropic behavior of the resin, mass uptake can be estimated from the ΔRH, measured by the free sensor as:

(21) $$m_{estimated}=3\;\rho_{H_{2}O}\;V_{0}\;\frac{K_{RH}^{resin}\Delta RH}{1-p_{e}}$$

Measured mass, estimated mass and relative error are reported in Figure 15. Error is evaluated as follows:

(22) $$err_{\%}=\frac{m_{measured}-m_{estimated}}{m_{measured}}\times 100$$

The absorption process, considered so far, consists of an accelerated procedure of 20 days, simulating years-long exposure to a hygrothermal environment. For this reason, it is sound to assume that, during transient, the sensor located inside the micro-perforated capillary tube lags behind the resin; as a matter of fact, the sensor always underestimates the mass of moisture absorbed. At saturation, the system reaches its equilibrium, bringing the error to zero and so allowing one to estimate the correct mass value. In real life, it is reasonable to assume that the change in moisture absorption is slower and therefore the sensor is always in hygroscopic equilibrium with the component.

## 5. Conclusions

In recent years, moisture uptake, temperature and mechanical effects have gained significant attention due to the serious impacts these factors have on composite materials. Consequently, SHM and HUMS have become essential for mitigating these issues by monitoring the health of composite structures and optimizing maintenance schedules, while also introducing new techniques in the process. Optical fibers with FBG sensors play a crucial role in this field, thanks to their low cost, high accuracy, high sensitivity, small size and real-time monitoring capabilities. These sensors allow for the monitoring of temperatures and strains, as well as for the detection of relative humidity, which alters the sensor’s response due to its interaction with the fiber coating. However, the challenge of decoupling each quantity, which affects the sensing region, remained an ongoing area of research. This study addresses this limitation through the development of an innovative sensor system supported by a modified Bragg equation, specifically designed for coated optical fiber embedded in a resin system subjected to hygrothermal-mechanical loads. Additionally, this method provides a means to correct data inconsistencies in already installed sensors. In this study, three FBG sensors embedded in a resin specimen were used to develop an innovative device capable of decoupling mechanical, thermal and hygroscopic effects. The first FBG sensor is sensitive exclusively to temperature, since it is inscribed on an uncoated optical fiber, making it insensitive to moisture absorption and enclosed within a metallic capillary tube, which renders it insensitive to external mechanical deformations. The second sensor is encapsulated into a micro-perforated capillary and is influenced by both temperature and relative humidity. From a mechanical point of view, it is isolated from external forces due to the presence of the capillary tube. However, it remains sensitive to moisture absorption thanks to the holes in the capillary wall, which allow moisture to reach the sensor. The third sensor is directly embedded in the resin and is affected by temperature, relative humidity and mechanical strain. In summary, the first sensor measures temperature, the second sensor provides both temperature and relative humidity values and the third sensor measures temperature, relative humidity and mechanical strain on the fiber. This configuration enables the formulation of a system of linearly independent equations, allowing for the decoupled calculation of the values of temperature (ΔT), relative humidity (ΔRH) and mechanical strain (Δε).

The innovative configuration of sensors, supported by the development of a modified Bragg law, provides an accurate framework for characterizing the combined hygrothermal-mechanical effects acting on FBGs embedded in bulk resin. The modified Bragg law incorporates the resin’s contribution, exploiting assumptions of small displacements, linear elasticity, perfect fiber/coating/resin adhesion and superposition of effects. This formulation accurately describes the behavior of FBGs embedded in bulk resin when subjected to hygrothermal-mechanical loads, with an error lower than 5%. The innovative device accurately detects and decouples temperature, relative humidity and mechanical strains by means of an independent system of FBG sensors. The proposed system also enhances predictive maintenance models by improving the precision of HUMS and SHM systems. In aeronautical applications, composite materials such as CFRP or GFRP are often used, where the fiber is hygrophobic and the matrix, typically epoxy-based, is hygroscopic. The presence of moisture within the composite primarily affects the matrix phase, as moisture acts as a plasticizer, disrupts Van der Waals bonds, breaks cross-links within the polymeric chains and induces leaching issues. By quantifying the moisture absorbed by the matrix, it is possible to calculate the degradation of the mechanical properties of the matrix phase and, consequently, through mixing rules, the degradation of the mechanical properties of the composite material.

Overall, this study significantly advances FBG-based monitoring systems, improving their precision, accuracy and usefulness in Structural Health Monitoring (SHM) and Health and Usage Monitoring Systems (HUMSs). The methodology enables the reliable decoupling of critical parameters, allowing the prediction of long-term performance and resilience of composite structures, particularly in aerospace applications where moisture-induced degradation is a critical concern. Furthermore, these findings establish a foundation for the development of improved calibration techniques and protective strategies in composite materials, promoting durability and safety during service. Such advancements would strengthen the role of FBG sensors as indispensable tools in SHM systems, ensuring enhanced reliability and performance throughout the service life of critical components.

## 6. Patents

The present work has been submitted for filing at the patent office, with application number 102024000013114, and is currently under review.

## Figures and Tables

**Table 1:** System’s parameters: subscript “*g*” refers to the glass, subscript “*c*” to the coating and subscript “*r*” to the resin system.

Parameter	Symbol	Value
Core + cladding radius	$r_{g}$	62.5 μm
Coating radius	$r_{c}$	77.5 μm
Resin section area	$A_{r}$	34.44 mm^2^
Glass Young modulus	$E_{g}$	70 GPa
Coating Young modulus	$E_{c}$	2.5 GPa
Resin Young modulus	$E_{c}$	3.08 GPa
Photo-elastic coefficient	$p_{e}$	0.2126 [-]
Thermo-optic coefficient	ξ	$5.81\times 10^{-6}\;1/K$
Glass CTE	$\alpha_{g}$	$1.020\times 10^{-6}\;1/K$
Coating CTE	$\alpha_{c}$	$3.636\times 10^{-5}\;1/K$
Resin CTE	$\alpha_{r}$	$1.235\times 10^{-4}\;1/K$
Coating swelling coefficient	$\beta_{c}$	$5.589\times 10^{-5}\;1/\mathrm{RH}\%$
Resin swelling coefficient	$\beta_{r}$	$1.636\times 10^{-4}\;1/\mathrm{RH}\%$

## Data Availability

The data presented in this study are available on request from the corresponding author.

## Abbreviations

The following abbreviations are used in this manuscript:

CFRP	Carbon Fiber Reinforced Polymer
CoV	Coefficient of Variance
CTE	Coefficient of Thermal Expansion
FBG	Fiber Bragg Grating
FO	Fiber Optic
GFRP	Glass Fiber Reinforced Polymer
HUMS	Health and Usage Monitoring System
RVE	Representative Volume Element
SHM	Structural Health Monitoring
